# Case Report: Ultrasonographic and computed tomographic features of presumed gastric plasmacytoma (plasma cell tumor) with ulceration in two dogs

**DOI:** 10.3389/fvets.2025.1634049

**Published:** 2025-08-12

**Authors:** Seoro Park, Soyoung Jeung, Yewon Ji, Matti Kiupel, Kichang Lee, Hakyoung Yoon

**Affiliations:** ^1^Department of Veterinary Medical Imaging, College of Veterinary Medicine, Jeonbuk National University, Iksan, Republic of Korea; ^2^VIP Animal Medical Center, Dongsomun-ro, Seongbuk-gu, Seoul, Republic of Korea; ^3^Veterinary Diagnostic Laboratory, Michigan State University, Lansing, MI, United States; ^4^Antech Asia, Kowloon, Hong Kong SAR, China

**Keywords:** canine, gastric tumor, plasmacytoma, ultrasound, computed tomography

## Abstract

Gastric plasmacytoma is rare in dogs, with only four previous reports providing limited descriptions of abdominal ultrasound (AUS) and computed tomography (CT) findings. This study presents the AUS and CT imaging features of two histopathologically suspected gastric plasmacytomas. Combining the four previous and two new cases, these tumors predominantly occurred in the greater curvature and pyloric antrum as solitary round masses connected to the submucosal or transmural layers. Pre- and post-contrast tissue attenuation (Hounsfield unit; HU) differences exceeded 40, with variable AUS echogenicity and echotexture. Imaging features may resemble those of spindle cell tumors, requiring histological and immunohistochemical examinations for definitive diagnosis.

## 1 Introduction

Gastric tumors are rare among all canine neoplasms, and plasmacytomas are even more uncommon, accounting for less than 1% of all cases (1, 2). Adenocarcinoma is the most common, followed by spindle cell tumors, such as leiomyoma, leiomyosarcoma, and gastrointestinal stromal tumor (GIST) (1, 3–6). Other rare cases include mast cell tumors, histiocytic sarcomas, and plasmacytomas (2, 7–11). Spindle cell tumors predominantly occur in the fundus, whereas adenocarcinoma predominantly occurs in the gastric body and pyloric antrum (12–14). Clinical signs are often nonspecific, with vomiting being the most common, followed by weight loss, anorexia, melena, diarrhea, and abdominal pain (15). Due to this nonspecificity, histopathological confirmation, particularly through supplementary immunohistochemistry, is indispensable for achieving an accurate diagnosis.

Although histopathology remains essential for diagnosis, imaging is highly useful in the evaluation of tumors. Abdominal ultrasonography (AUS) offers a rapid and noninvasive means of screening for suspected gastric tumors. In contrast, contrast-enhanced computed tomography (CT) provides more comprehensive information, including vascular dynamics of the mass and changes such as mucosal erosion, ulceration, hemorrhage, and mineralization. CT is also instrumental in assessing metastatic status and the involvement of other abdominal organs. Thus, integrating various imaging modalities facilitates a more thorough assessment of gastric tumors. Despite this, imaging features specific to gastric plasmacytomas in dogs have not been well characterized. In this report, we describe the sonographic and CT features of two canine cases with gastric plasmacytoma.

## 2 Case description

### 2.1 Case 1

A 9-year-old, 3.5-kg male Pomeranian dog presented with a 2-day history of vomiting and anorexia. Physical examination revealed lethargy, with no evidence of increased abdominal pressure upon palpation. Hematologic evaluation indicated mild anemia (hematocrit 22.5%, reference range: 37.3–61.7%), elevated C-reactive protein (>200 mg/L, reference range: 0–20 mg/L), mild electrolyte disturbances (Na^+^ 137 mmol/L, reference range: 145–151 mmol/L; K^+^ 3.07 mmol/L, reference range: 3.9–5.1 mmol/L; Ca^+^ 1.08 mmol/L, reference range: 1.16–1.40 mmol/L; Cl^−^ 96 mmol/L, reference range: 110–119 mmol/L), and metabolic alkalosis (pH 7.56, reference range: 7.31–7.46; HCO_3_^–^ 30.1 mmol/L, reference range: 17–28 mmol/L).

Abdominal radiography (R-400-125, VETTER-DR9, Korea; 62 kVp, 200 mA) was performed. A lateral abdominal radiograph identified 3.7 × 4.7 mm round structures with soft tissue opacity in the pyloric region, raising suspicion of a pyloric mass or pyloric outflow tract dilation (Supplementary Figure S1A). Multiple mineral opaque structures, measuring approximately 1.0 × 2.5 cm (length × height), similar to those observed in the intestinal segments, were also observed.

This suggested gastrointestinal ingestion; however, dystrophic calcification within the pyloric mass was also considered (Supplementary Figure S1B).

**Figure 1:**
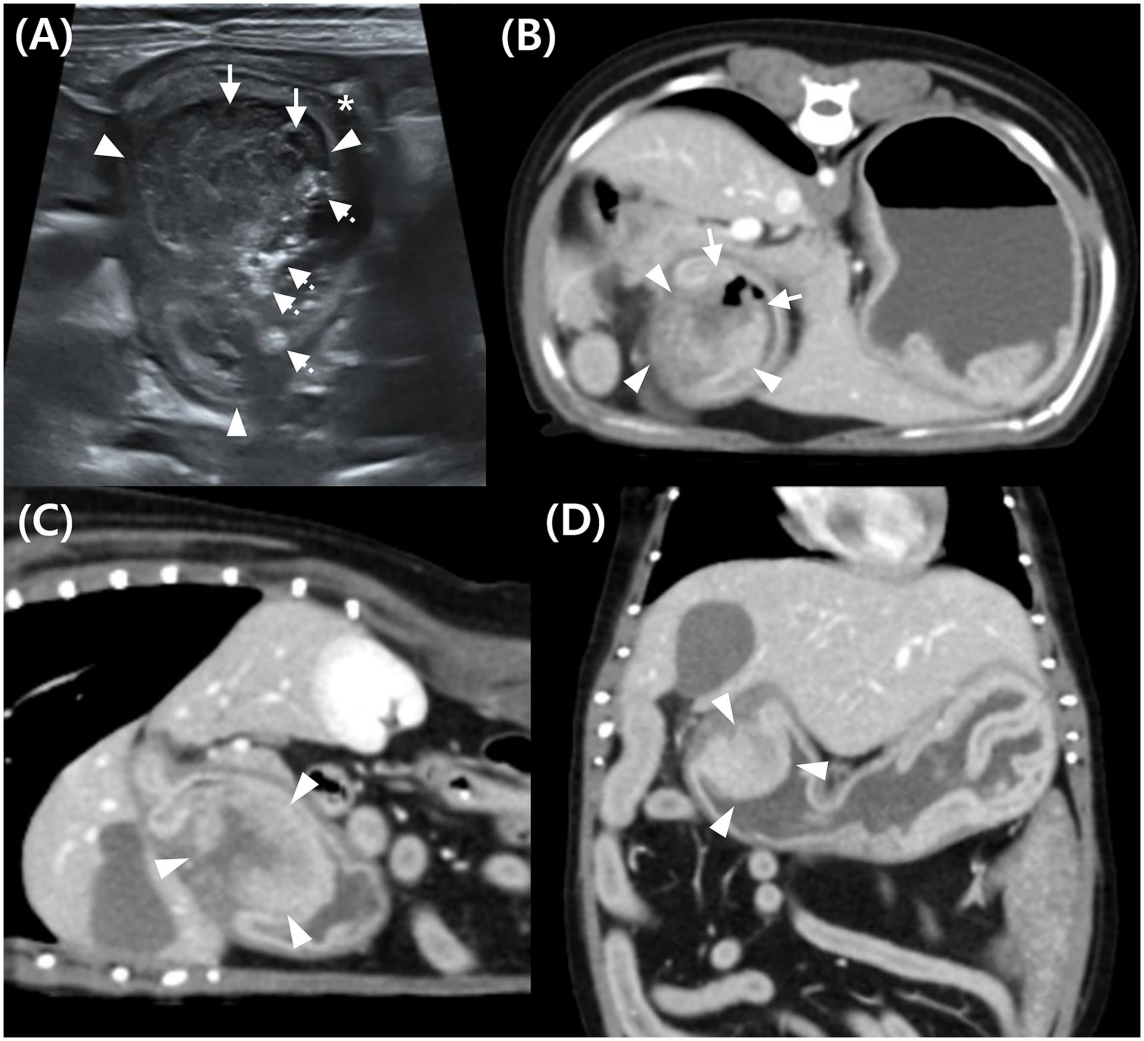
Abdominal ultrasound **(A)** and post-contrast computed tomography (CT) images **(B–D)** of a mass in the pyloric antrum in Case 1. **(A)** On ultrasound, a mixed echogenic mass (arrowhead) attached to the gastric wall, narrowed pyloric lumen (*), hypoechoic area indicating necrosis or hemorrhage (arrow), and hyperechoic content suggestive of ingesta (dotted arrow). **(B–D)** Post-contrast CT images of the mass (arrowhead) in transverse **(B)**, sagittal **(C)**, and dorsal **(D)** planes. The mass (arrowhead) shows continuity with both inner and outer gastric wall layers, suggesting transmural involvement, with heterogeneous contrast enhancement. Disrupted mucosal continuity in the pyloric region (arrow) suggests ulceration.

Ultrasonography was performed using a linear i18LX5 probe (12 MHz), with the dog in dorsal recumbency (Aplio i700, Canon Medical Systems, Tustin, CA, United States). Abdominal ultrasound (AUS) revealed an approximately 2.01 × 1.77 cm oval-shaped mass in the pyloric antrum region of the stomach (Figure 1A). The mass parenchyma exhibited heterogeneous echogenicity, with a 2-mm hypoechoic area, which showed no distinct blood flow on Color Doppler ultrasound, suggesting necrosis or hemorrhage. Partial loss of wall layering was observed, and the mass appeared to be connected to the submucosa or muscularis, suggesting that it originated from these layers. The gastric wall in the pyloric antrum (from the mucosa to the serosa) measured up to 3.3 mm, with no evident thickening. Similarly, no significant wall thickening was observed in the fundus or gastric body. The mass caused narrowing of the pyloric antrum and intragastric fluid retention, raising suspicion of partial obstruction on imaging, potentially related to the observed vomiting. Enlargement of the pancreaticoduodenal lymph nodes (LNs) and increased echogenicity of the adjacent peritoneal fat indicated possible lymphadenitis with peritonitis or LN metastasis from the gastric mass.

Computed tomography (CT) was performed using a 16-slice helical CT scanner (Brivo CT 385; GE Medical Systems, United States) under general anesthesia with the dog positioned in ventral recumbency. The CT scan was conducted using the following parameters: 120 kVp, 85 mAs, 512 × 512 matrix, 2.5 mm slice thickness, 0.75 s rotation time, and 0.938 collimation beam pitch. For contrast-enhanced CT, iohexol (Omnihexol 300; Korea United Pharmaceutical, Seoul, Korea) was manually injected into the cephalic vein at a dose of 600 mg iodine/kg. Post-contrast images were acquired with a 90-s delay. The CT images were evaluated using an abdominal window [window level = 40 Hounsfield units (HU); window width = 400 HU]. Based on previous reports, gastric wall layers were evaluated on CT by categorizing them into a strongly enhancing inner layer (representing the mucosa and submucosa) and a less-enhancing outer layer (representing the muscularis and serosa) (14).

Pre-contrast CT revealed a hyperattenuating foreign body in the stomach, measuring approximately 1.6 × 0.6 × 3.5 cm (length × height × width). It appeared as an aggregation of multiple 2–4 mm structures and did not cause gastric obstruction. The object, located in the gastric body, was removed during pylorectomy and confirmed to be a fragmented, partially digested chicken bone treat. CT imaging revealed a contrast-enhancing, hyperattenuated, oval-shaped mass with well-defined margins, measuring approximately 2.83 × 2.06 × 2.37 cm (length × height × width) (Figures 1B–D). Assessment of the HU values of mass parenchyma revealed a difference >10 HU between maximum and minimum attenuation values, consistent with heterogeneous contrast enhancement (16, 17). The region of interest (ROI) was defined by placing 10 circular areas, each approximately 2 mm in diameter, within the lesion for assessment. The average HU values pre- and post-contrast were 43.06 and 123.94, respectively, determined by placing the largest ROI within the lesion, obtaining three measurements, and calculating the mean (14).

The peripheral portion of the mass demonstrated continuity with the inner layer of the gastric wall, which was contrast-enhanced and appeared as a hyperattenuating line with a similar attenuation value (Figures 1B–D). The central portion of the mass appeared relatively hypoattenuating and was continuous with the outer layer of the gastric wall. These findings suggest that the mass was transmural.

Gas-attenuating structures were observed in areas where mucosal continuity was disrupted, indicating possible mucosal erosion or ulceration (Figure 1B). Fat stranding in the adjacent peritoneal fat suggested potential peritonitis or mass invasion into the peritoneum. Enlargement of the gastric, pancreaticoduodenal, hepatic, and splenic LN was observed.

Based on these imaging findings, the provisional diagnoses included spindle cell tumors, such as leiomyoma, leiomyosarcoma, or GIST, potentially originating from the submucosa or muscularis. Additionally, epithelial tumors, such as adenoma and adenocarcinoma, as well as round-cell tumors, such as lymphoma, could not be ruled out. Concurrently, suspected mucosal erosion or ulceration in the pyloric region, enlargement of some intraperitoneal LNs, and localized peritonitis in the right upper abdomen were observed.

**Figure 2:**
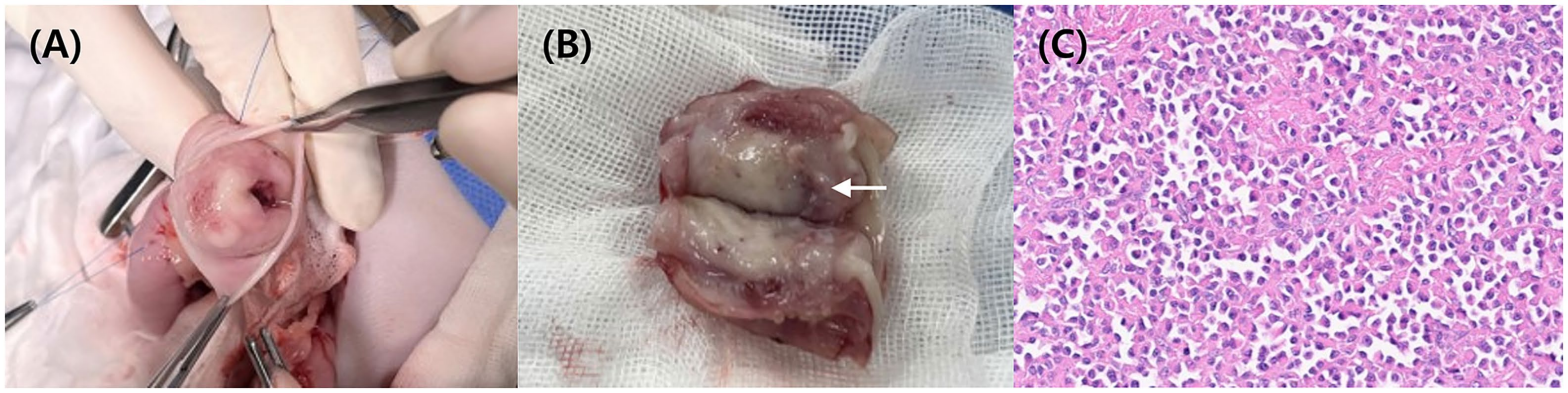
Intraoperative image **(A)** showing an incision in one wall of the pylorus. Image of the resected pylorus **(B)** reveals a smooth, white luminal surface. Grossly, the reddish area (arrow) was suspected to represent an erosion or ulcerative lesion. Histopathology **(C)** confirms the presence of numerous plasma cells in the submucosa layer HE.

Eight days after CT imaging, pylorectomy was performed to remove the pyloric mass. Following laparotomy, vascular isolation was performed near the pylorus. No distinct adhesions between the pyloric mass and the adjacent peritoneum were observed. The pylorus was resected with a 2-cm margin around the mass, and the antrum and proximal duodenum were sutured (Figure 2A). The resected pyloric mass had smooth margins and a white-to-pink color (Figure 2B). Fine-needle aspiration (FNA) and histopathology were performed on the tissues. FNA revealed numerous round cells, which were later confirmed to be plasma cells on histopathological examination of the excised mass. Histological examination showed a non-encapsulated, densely cellular, well-circumscribed, expansile, nodular mass in the submucosa extending into the mucosa and muscularis. The tumor consisted of round cells with abundant amphophilic cytoplasm and hyperchromatic, round-to-oval nuclei. Occasional multinucleated cells and rare mitotic figures were observed (Figure 2C). The lesion was histologically consistent with a plasmacytoma. Tumor cells infiltrated up to the resection margin, and the serosal surface was not identified in the submitted tissue. Over a 12-day hospitalization period, no gastrointestinal symptoms were noted, and appetite and vitality remained normal. No clinical signs were reported after discharge.

### 2.2 Case 2

A 10-year-old, 3.6-kg female Maltese dog presented with abdominal and cardiac AUS monitoring due to elevated liver enzymes and myxomatous mitral valve disease (ACVIM stage B1-B2). Physical examination revealed a grade 3/6 murmur with no other abnormalities. Blood tests showed elevated alkaline phosphatase (892 U/L, reference range, 47–254 U/L) and alanine aminotransferase (103 U/L, reference range, 17–78 U/L), while C-reactive protein (9 mg/L, reference range, 0–9 mg/L) was within normal limits. Elevated liver enzymes had been consistently above normal since the first visit 3 years prior, with no significant changes observed to date, and an abdominal ultrasound was recently performed to evaluate the current condition.

Abdominal radiography (HF-525 PLUS, ECORAY, Seoul, Korea; 70 kVp, 200 mA) was performed in the right lateral and ventrodorsal positions. Mild hepatomegaly was suspected in the right lateral view, but no mass opacities were observed.

**Figure 3:**
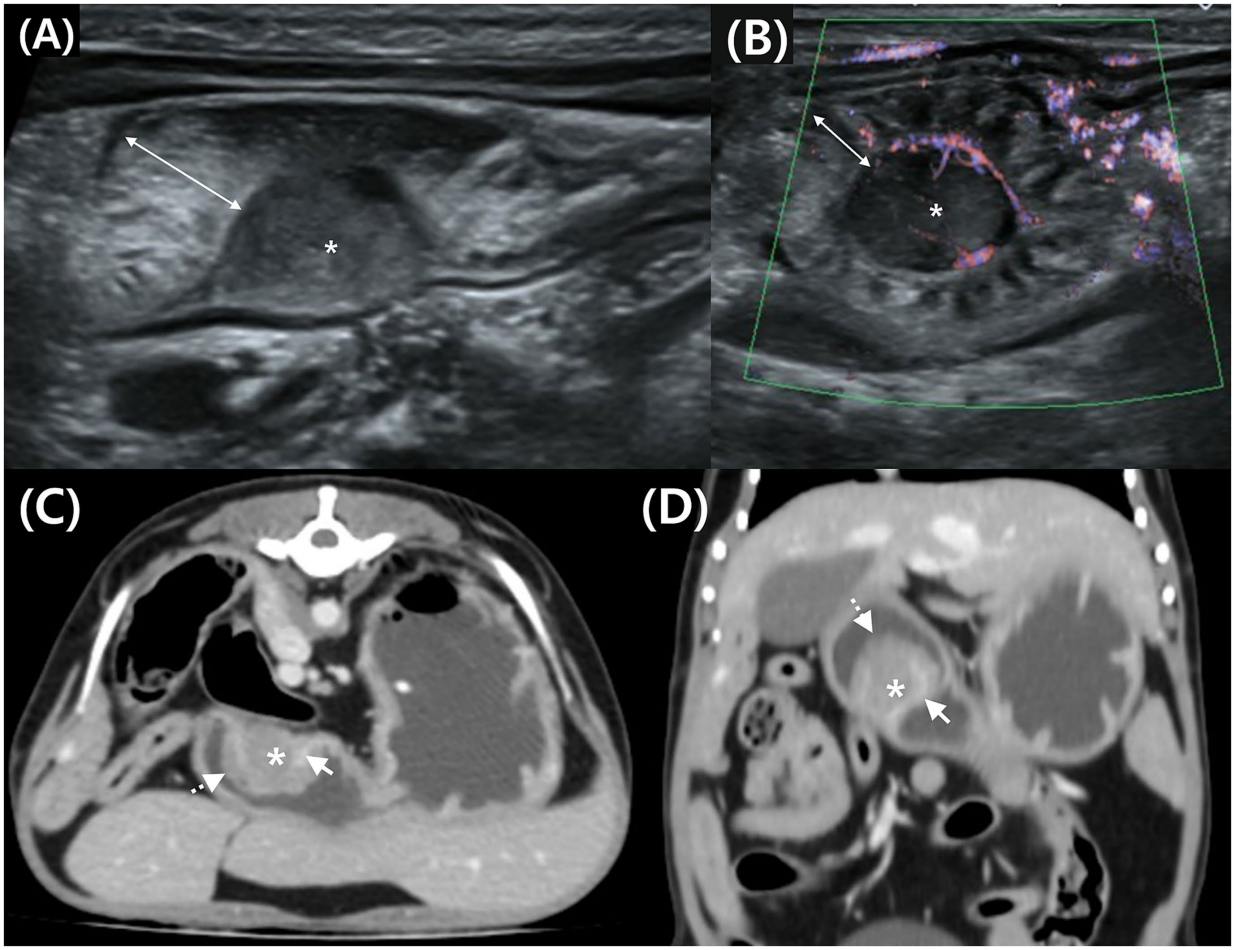
Transverse **(A)** and longitudinal **(B)** abdominal ultrasound (AUS) images, and transverse **(C)** and dorsal **(D)** post-contrast CT images of a mass in the gastric body in Case 2. **(A)** Hypoechoic mass (*) in the submucosal layer and thickened mucosa (double arrow) on the luminal side. **(B)** Peripheral blood flow is detected at the mass margin using advanced dynamic flow Doppler. **(C,D)** Post-contrast CT shows a mass (*) compressing the enhancing inner layer (arrow) and thickened mucosa (dotted arrow) on the luminal surface.

An additional AUS examination (Aplio i800, Canon Medical Systems, Europe BV, 13 MHz, linear-array probe) was performed. AUS revealed a homogeneous, hypoechoic, round-to-oval mass measuring approximately 1.01 × 1.77 cm, located in the gastric body (Figure 3A). Advanced dynamic flow (ADF) color Doppler imaging demonstrated peripheral microvascularity, confirming the presence of blood flow at the lesion margin (Figure 3B). This region was presumed to correspond to the mucosa or submucosa, where vascularity is most prominent, and the mass appeared to originate in submucosa. The mass was surrounded by a thick mucosal layer.

CT was performed using the same procedure as described in the previous case. Similar to the AUS findings, a round mass approximately 1 cm in size was identified in the greater curvature of the gastric body (Figures 3C, D). Post-contrast images showed enhancement at the interface between the mass and gastric wall. The mass was observed external to the inner gastric layer, which remained relatively intact, suggesting a possible origin from the outer layers—specifically the muscularis, serosa, or adjacent submucosa. Thickening of the luminal mucosa was also observed.

Analysis of the HU values of the mass parenchyma revealed a difference of <10 between the maximum and minimum values, indicating homogeneous contrast enhancement (Figures 3C, D). The ROI settings were consistent with those in Case 1. The mean HU values of the mass parenchyma were 49.58 pre-contrast and 95.09 post-contrast, with ROI settings and HU evaluation consistent with those of Case 1.

Mild enlargement of nearby gastric, hepatic, jejunal, and colic LNs was observed. A provisional diagnosis of spindle cell tumors, such as leiomyoma, leiomyosarcoma, or GIST, was primarily considered. However, other gastric neoplasms, including adenoma, adenocarcinoma, and lymphoma, could not be excluded.

**Figure 4:**
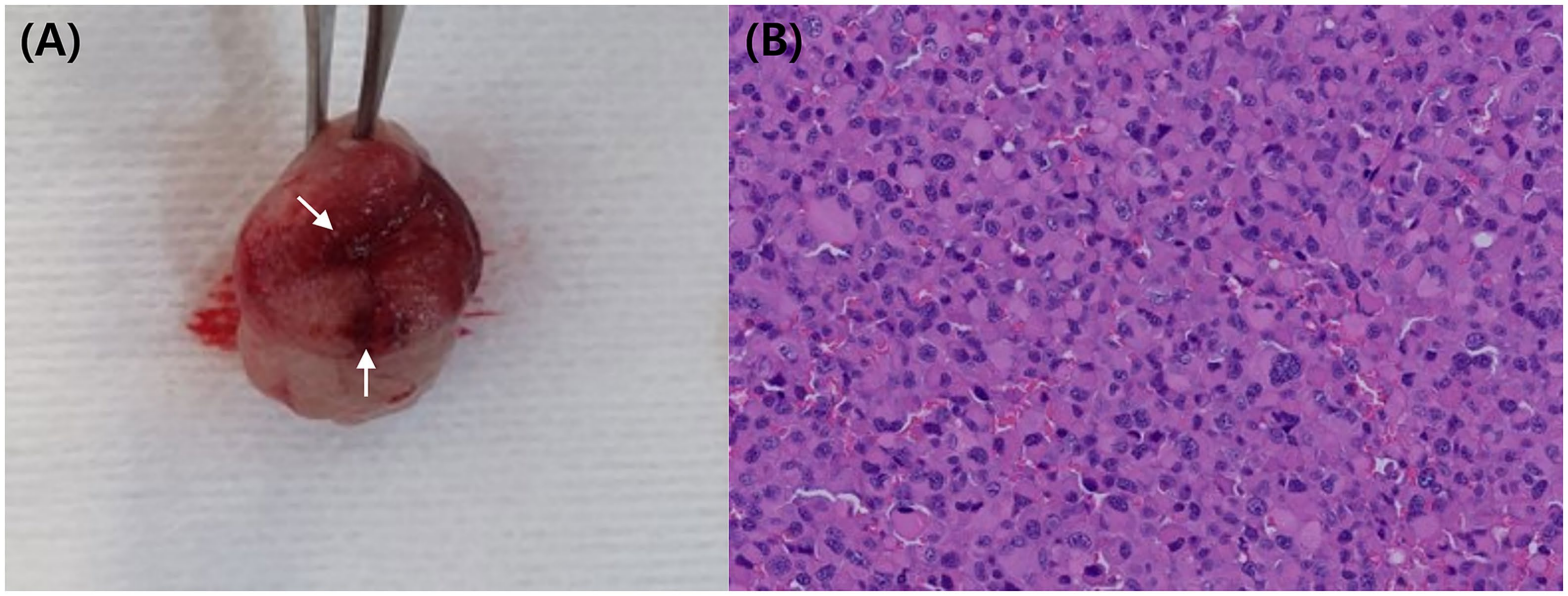
Resected mass **(A)** following partial gastrectomy, showing a thickened wall and narrowed lumen. Grossly, the reddish area (arrow) was suspected to represent an erosion or ulcerative lesion. Histopathology **(B)** confirmed the presence of numerous plasma cells in the submucosa layer HE.

A partial gastrectomy was performed to remove the mass in the greater curvature of the stomach. Grossly, the wall appeared locally thickened, narrowing the lumen, and an area suspicious for erosion or ulceration was identified (Figure 4A). Histological examination revealed a plasmacytoma extending across the gastric mucosa, muscularis mucosae, and submucosa. Tumor cells exhibited round nuclei with coarse chromatin, 1–2 distinct nucleoli, abundant eosinophilic cytoplasm, and clear cell borders. Marked variations in cell and nuclear size were evident, with frequent multinucleation and enlargement of nuclear cells (Figure 4B). Additionally, 1–2 mitotic figures per high-power field were observed. The mucosal surface was ulcerated, covered with fibrin, and contained degenerated neutrophils. The resection margins (submucosa and mucosa) showed a clearance ranging from 1 mm to 6.4 mm, indicating complete excision. This lesion, a rare gastrointestinal plasmacytoma, may be clinically associated with monoclonal gammopathy and local LN metastasis (6, 18). No complications were observed during a 7-d hospitalization or follow-up period after discharge.

## 3 Discussion

To date, only six cases of gastric plasmacytoma have been reported, with the imaging characteristics described in only four cases. However, even these four reports did not provide detailed AUS or CT findings (4–7). We aimed to specifically describe the AUS and CT imaging features of two gastric plasmacytoma cases and compare their characteristics to those of previously reported canine cases.

In the four previously reported cases, patient ages ranged from 8 to 12 years, placing them primarily in the middle-aged group (4–7). The breeds varied and included mixed breeds, Shih Tzu, and Jack Russell Terrier. The most common clinical sign was vomiting, accompanied by hematemesis, diarrhea, melena, lethargy, and anorexia. In three cases, imaging revealed gastric plasmacytomas in the pyloric antrum (*n* = 2) and greater curvature (*n* = 1). Morphologically, a solitary round mass was observed in two cases, while a crateriform mass was noted in one case. Submucosa, muscularis, and serosa were involved in one case. Echogenicity was described as hypoechoic in two cases, with heterogeneous echotexture reported in one. LN metastasis was confirmed in one case (4), with no evidence of metastasis to other organs.

The two newly reported cases included gastric plasmacytomas in the pyloric antrum (Case 1) and greater curvature (Case 2). Both presented as solitary round masses, distinct from the transmural thickening typically observed in adenoma, adenocarcinoma, and lymphoma (13). In both cases, imaging suggested connectivity with the submucosal and muscularis layers.

The marked pre- and post-contrast HU differences observed in the present cases contrast with the lower enhancement typically seen in gastric lymphomas; however, further cases are needed to determine whether this distinction can be used diagnostically (13). The approximately 37-unit difference between the two cases likely reflects variables such as post-contrast imaging timing or heart rate, as well as the predominantly affected layer (submucosa or muscularis).

In Case 1, the submucosa was the primary involved area, with extension into the mucosa and muscularis. In Case 2, tumor cells were observed across the mucosa, submucosa, and muscularis layers. In both cases, the tumor cells infiltrated the adjacent layers, making a definitive origin difficult to determine. However, when considering these two new cases alongside previously reported ones, the submucosa and muscularis appear more likely to be the primary origin sites of the tumor than the mucosa.

Contrast enhancement patterns differed between the cases: heterogeneous in Case 1 and homogeneous in Case 2. This difference is likely attributable to concurrent ulceration, necrosis, or hemorrhage in Case 1 rather than inherent differences in the mass parenchyma.

In summary, plasmacytomas are typically located in the greater curvature and pyloric antrum, appearing as solitary round masses in the submucosal or muscularis layers, with a pre- and post-contrast HU value difference exceeding 40 (Supplementary Figure S2). AUS echogenicity and echotexture can vary. While gastric plasmacytomas may resemble spindle cell tumors on imaging, differentiation can be achieved through histologic and immunohistochemical examination.

In Case 2, ulceration associated with gastric plasmacytoma was documented for the first time. In Case 1, CT suggested a possible ulcerative lesion, but this was not confirmed by histopathology. In human cases of plasmacytoma, ulceration may result from localized inflammatory responses and tissue necrosis induced by the tumor itself (8). Partial obstruction of the pyloric antrum leading to intragastric food retention could have contributed to ulceration (9). And, in human plasmacytomas, the M protein is known to cause hemostatic disorders; however, this has not been investigated in these cases. If canine plasmacytomas function similarly, this could have accelerated pre-existing mucosal damage and ulcer progression (10, 11).

The limitations of this study include the lack of FNA and histopathology of enlarged adjacent LNs, liver, and spleen, limiting precise evaluation of metastasis or invasion extent. Additionally, the absence of arterial-phase CT imaging precluded the comparison of the three-phase contrast enhancement patterns. Finally, some plasmacytomas may exhibit a phenotype similar to mature B-cell lymphoma on histopathological examination, indicating that the possibility of B-cell lymphoma in the reported cases cannot be entirely ruled out (19). Differentiation via multiple myeloma oncogene 1 immunohistochemistry is possible, but further testing was not conducted (18, 20–22). Investigating these aspects in future studies could provide more comprehensive insights into the diagnostic and prognostic features of gastric plasmacytomas.

In conclusion, this study presents the AUS and CT imaging features of two histopathologically suspected gastric plasmacytomas. Although rare, gastric plasmacytomas should be considered in the differential diagnosis when a solitary round mass with notable contrast enhancement and connectivity to the outer layer of the greater curvature or pyloric antrum is observed. However, owing to their similarity to the imaging features of spindle-cell tumors, histological and immunohistochemical confirmation is essential.

## Data Availability

The original contributions presented in the study are included in the article/Supplementary material, further inquiries can be directed to the corresponding author.

## Supplementary material

SUPPLEMENTARY FIGURE S1 Lateral **(A)** and ventrodorsal **(B)** abdominal radiographs of Case 1. A round soft tissue structure (arrowhead) is observed on the lateral view in the ventral aspect of the upper abdomen. Multiple radiopaque structures (arrows) are noted in the same region and within the intestine.

SUPPLEMENTARY FIGURE S2 Dorsal plane computed tomography (CT) images showing: **(A)** a previously reported gastric plasmacytoma in a 12-year-old Jack Russell Terrier (post-contrast, heterogeneous contrast enhancement) (6), **(B)** a mass in the pyloric antrum of Case 1 (post-contrast, heterogeneous contrast enhancement), and **(C)** a mass in the greater curvature of Case 2 (post-contrast, homogeneous contrast enhancement). In all three cases, the contrast-enhanced inner layer remains preserved, with the mass identified as a solitary round structure growing outward from the inner layer. Arrows indicate the mass location.
