# Supplementary figures and images for: Case Report: Ultrasonographic and computed tomographic features of presumed gastric plasmacytoma (plasma cell tumor) with ulceration in two dogs
Source: Front Vet Sci. 2025 Aug 12;12:1634049. doi: 10.3389/fvets.2025.1634049 (PMC12379048; doi:10.3389/fvets.2025.1634049)

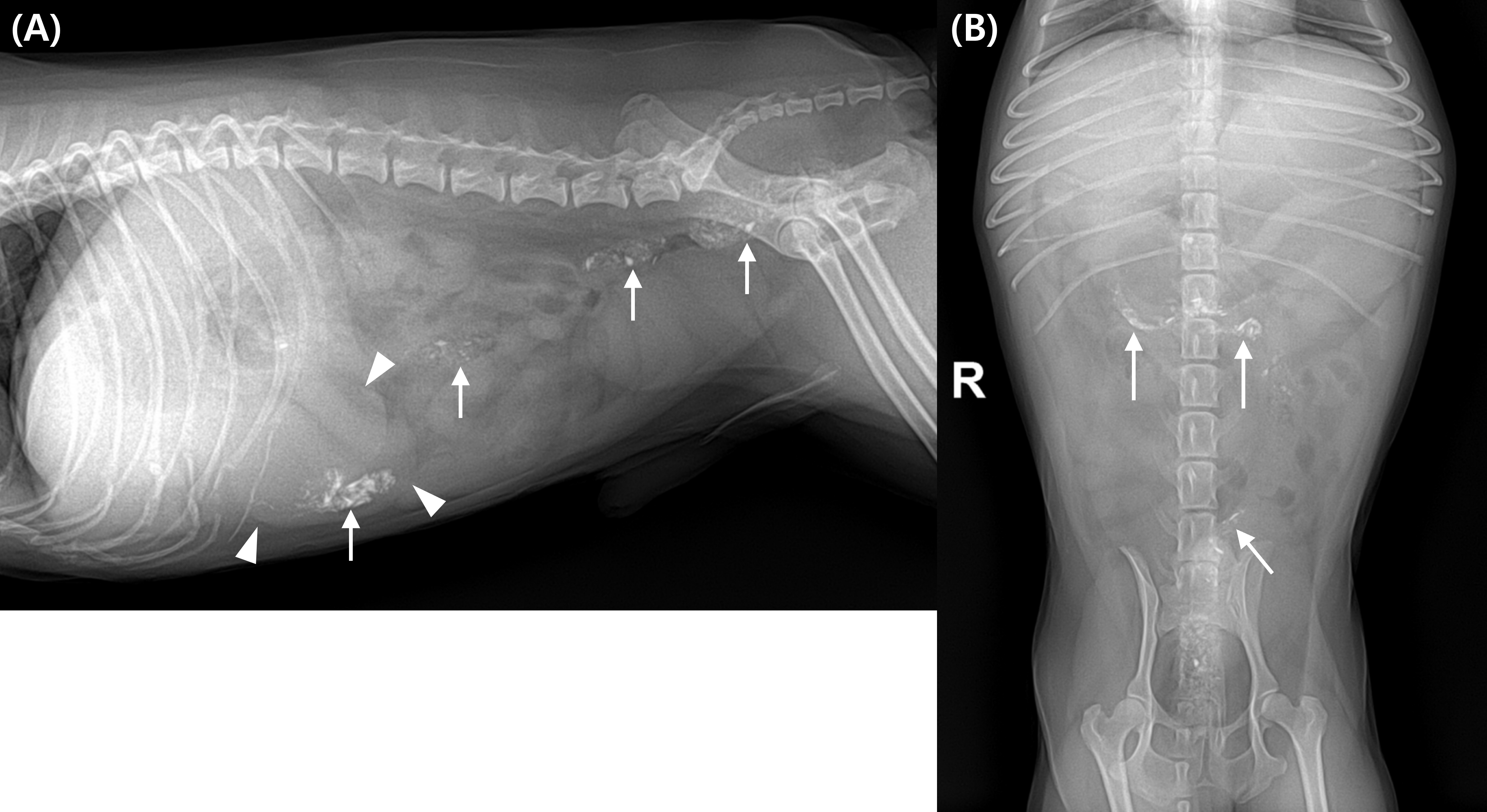

Supplement: SUPPLEMENTARY FIGURE S1 — Lateral (A) and ventrodorsal (B) abdominal radiographs of Case 1. A round soft tissue structure (arrowhead) is observed on the lateral view in the ventral aspect of the upper abdomen. Multiple radiopaque structures (arrows) are noted in the same region and within the intestine. [file Image_1.TIF]

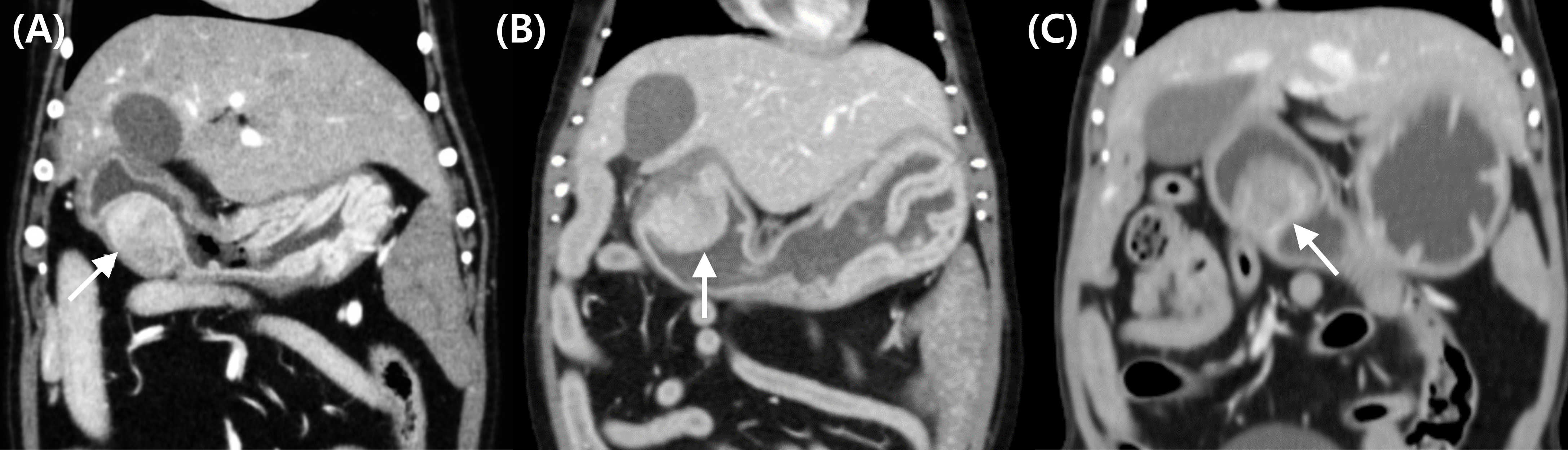

Supplement: SUPPLEMENTARY FIGURE S2 — Dorsal plane computed tomography (CT) images showing: (A) a previously reported gastric plasmacytoma in a 12-year-old Jack Russell Terrier (post-contrast, heterogeneous contrast enhancement) (6), (B) a mass in the pyloric antrum of Case 1 (post-contrast, heterogeneous contrast enhancement), and (C) a mass in the greater curvature of Case 2 (post-contrast, homogeneous contrast enhancement). In all three cases, the contrast-enhanced inner layer remains preserved, with the mass identified as a solitary round structure growing outward from the inner layer. Arrows indicate the mass location. [file Image_2.TIF]
